# Therapeutic effects of magnesium and vitamin B6 in alleviating the symptoms of restless legs syndrome: a randomized controlled clinical trial

**DOI:** 10.1186/s12906-022-03814-8

**Published:** 2022-12-31

**Authors:** Ali Jadidi, Alireza Rezaei Ashtiani, Ali Khanmohamadi Hezaveh, Seyed Mohamad Aghaepour

**Affiliations:** 1 School of Nursing, Arak University of Medical Sciences, Arak, Iran; 2 Traditional and Complementary Medicine Research Center, Arak University of Medical Sciences, Arak, Iran; 3 School of Medicine, Arak University of Medical Sciences, Arak, Iran; 4 Student Research Committee, Arak University of Medical Sciences, Arak, Iran

**Keywords:** Restless legs syndrome, Willis-Ekbom Disease, Magnesium, Vitamin B6, Pramipexole, Sleep disorders

## Abstract

**Background and objective:**

Restless legs syndrome/Willis-Ekbom Disease (RLS/WED) is one of the most prevalent sleep disorders. There are contradicting data about the effectiveness of magnesium and vitamin B6 in alleviating the symptoms of this condition. Therefore, this study aimed to assess the efficacy of magnesium and vitamin B6 in alleviating the symptoms of RLS/WED.

**Methods:**

A single-blind study was conducted on individuals with this illness for at least three months. Randomly, 75 patients were assigned into three groups: magnesium, vitamin B6, and placebo. The experimental group received daily doses of 40 mg vitamin B6 or 250 mg magnesium oxide. While others in the control group merely received a placebo. Patients’ disease severity and sleep quality were evaluated three times using standard questionnaires (at the beginning of the study, one and two months after therapy). Utilizing SPSS22 software and the ANOVA, t-test, and repeated measure tests, statistical analysis was conducted.

**Results:**

The mean and standard deviation of sleep quality and disease severity at the beginning of the trial and throughout the first month following the intervention did not differ statistically between the three groups. In the second month following the intervention, the mean and standard deviation of sleep quality and disease severity were significantly different (*P* = 0.001).

**Conclusion:**

Taking magnesium and vitamin B6 supplements can reduce the severity of symptoms of RLS/WED patients and improve their sleep quality.

## Introduction

Restless Legs Syndrome/Willis-Ekbom Disease (RLS/WED) is a sensory-motor condition characterized by aberrant sensations in the legs. Patients frequently experience a strong desire to move the afflicted limbs, and these strange sensations are represented partially or entirely by voluntary movements like walking [1]. These symptoms are frequently aggravated during sleep and frequently result in sleep disturbance. The condition might cause daytime sleepiness or weariness due to insufficient nighttime sleep. Other implications of this condition include a decline in quality of life, sadness, and anxiety disorders [2]. This issue is also recognized as a cardiovascular disease risk factor [3]. The disease prevalence is 7–10% among the general population and 20–30% among people with diabetes [4]. There are two types of the disease: idiopathic and secondary. Pregnancy, uremia, iron insufficiency, diabetes, and neuropathy are considered risk factors for the condition in its secondary form; nevertheless, the mechanism of the disease is not yet fully understood [1, 5].

RLS/WED is frequently misdiagnosed and inadequately treated. The initial step in treating this condition is to eradicate its underlying causes, such as iron deficiency anemia. Drug groups such as benzodiazepines, opioids, dopamine agonists, carbamazepine, gabapentin, pregabalin, and clonazepam are frequently used to treat RLS/WED [6].

Magnesium may have a role in the pathophysiology of RLS/WED since studies have revealed that magnesium levels are lower in RLS/WED patients compared to healthy controls [7]. Some studies have also demonstrated that oral and intravenous magnesium can benefit these patients [8, 9]. In his study, Sinniah described a patient with RLS/WED who recovered entirely after taking magnesium sulfate intravenously [10]. A study by Yıldırım & Apaydın, as well as a study by Meta et al., both show that magnesium therapy can lower the intensity of symptoms of this illness and improve the quality of sleep of patients, suggesting that magnesium may be considered an appropriate replacement for the treatment of patients with RLS/WED [8, 11]. However, there is some evidence that magnesium is useless in treating this condition [12].

On the other hand, vitamin deficiency may also contribute to the disease’s etiology. Several investigations have demonstrated that these individuals had lower blood levels of B vitamins, particularly vitamin B6, than the control group [13]. Lemoine et al.‘s study that the combination of vitamin B6 and medicinal plants may be beneficial in mild-to-moderate insomnia [14]. The study by Peters et al. also showed that dietary intake of vitamin B6 correlated significantly with insomnia and sleep quality  [15]. Pyridoxine’s action as a coenzyme in tryptophan metabolism suggests an increase in brain serotonin levels which has been known to suppress REM sleep [16]. Also vitamin B6 appears to have strong influence on night awakenings reduction [17]. A study also recommends that vitamin B6 can relieve painful cramps in pregnant women  [18]. However, no study has evaluated the therapeutic efficacy of B vitamins in these individuals. Due to the paucity of studies in this field and the ambiguity surrounding the therapeutic role of supplements in this disorder, the present study sought to determine the role of magnesium and vitamin B6 in alleviating RLS/WED symptoms in patients referred to neurology clinics in Arak.

## Materials and methods

This study was a single-blind investigation of Arak University of Medical Sciences clinic patients with RLS/WED. Inclusion criteria were a 3-month history of the condition, age between 15 and 50, and the absence of risk factors including pregnancy, renal failure, uremia, diabetes, multiple sclerosis, and anemia. Patients were randomly assigned into magnesium, vitamin B6, and control groups. Sealed envelopes with computer-generated randomized numbers were used to allocate patients to groups. In addition to the medicine (Pramipexole), the experimental groups received one daily pill of vitamin B6 (40 mg) or magnesium oxide (250 mg). In addition to pramipexole, the control group received only a placebo. In this trial, blinding was achieved by removing the pills from their packaging and placing them in a different container so that the patient did not know what medication he was taking, but the physician and researchers did. In addition, the persons were arranged in groups such that they were not connected. This study assessed the therapeutic benefit of changing two questionnaire scores following medication therapy. Exclusion criteria included reluctance to continue the trial, pregnancy during the study, and adverse medication reactions. Patients filled out the survey questionnaires three times (at the beginning of the study, one and two months after medication therapy).

This investigation utilized the International Restless Legs Scale (IRLS) and the Petersburg Sleep Quality Index (PSQI were utilized in this investigation. The IRLS consists of 10 items that are scored on a Likert scale ranging from 0 to 4. 0 signifies mild intensity, 11–20 moderate, 21–30 severe, and 40 − 31 very severe [19]. The symptom severity rating scale was validated by Walters et al. [20]. The PSQI assesses the individuals’ attitudes towards sleep quality in the past four weeks. This week consists of seven scales, where each scale’s score is between 0 and 3, and a score of 3 on each scale denotes the maximum negative. The overall score of this questionnaire is from 0 to 21, and a score of 6 or more indicates insufficient quality [21]. Multiple research has proven these surveys’ reliability and validity. After data collection, statistical analysis was conducted using SPSS 22 and ANOVA, t-test, and repeated measures.

## Results

In this study, 75 patients with RLS/WED were divided into three groups: control, magnesium oxide intervention, and vitamin B6 intervention. The independent t-test revealed that the mean age of the three groups did not differ substantially. The Chi-square test also revealed that other demographic characteristics did not differ significantly between the three groups (Table 1).

**Table 1:** Comparison of the demographic variables between study groups

Variables		Control	Vitamin B6 intervention	Magnesium intervention	*P*-Value
		Mean(SD)			
	Age	39.9(9.8)	38.7(8.7)	41.6(7.5)	0.89^*^
		**Frequency(%)**			
Gender	Male	6(24)	7(28)	5(20)	0.74^**^
	Female	19(76)	18(72)	20(80)	
Marital status	Married	20(80)	22(88)	22(88)	0.65^**^
	Single	5(20)	3(12)	3(12)	
Education	Primary school	4(16)	3(12)	4(16)	0.23^**^
	diploma	9(36)	8(32)	8(32)	
	Academic	12(48)	14(56)	13(52)	
Profession	Housewife	17(68)	15(60)	15(60)	0.37^**^
	Employee	3(12)	4(16)	3(12)	
	Worker	2(8)	3(12)	5(20)	
	Others	3(12)	4(16)	2(8)	

^*^ANOVA, ^**^Chi-squared test

The ANOVA test revealed that the mean PSQI score at the beginning of the intervention and one month after the intervention was not significantly different between the three groups, but there was a significant change (P 0.05) after two months. The IRLS questionnaire reveals the disease’s severity. At the start of the intervention and one month later, there was no significant difference between the three groups; however, after two months, there was a significant difference (Table 2).

**Table 2:** Comparison of sleep quality and severity of symptoms between groups in three measurement

Variables		Control	Magnesium intervention	Vitamin B6 intervention	*P*-Value
		Mean(SD)			
Sleep quality	Beginning of the study	16.6(2.95)	18.28(3.08)	18.4(2.95)	0.70^*^
	One month later	11.9(3.9)	10.8(3.1)	11.72(3.16)	0.65^*^
	Two month later	10.52(3.5)	5.92(2.59)	7.04(1.84)	0.001^*^
	*P*-Value	0.001^**^	0.001^**^	0.001^**^	
IRLS^#^	Beginning of the study	31.04(4.2)	30.96(7.71)	32.08(5.25	0.87^*^
	One month later	26.36(5.4)	21.84(4.53)	22.68(5.82)	0.55^*^
	Two month later	24.24(8.07)	16.08(5.88)	17.8(7.1)	0.001^*^
	*P*-Value	0.001^**^	0.001^**^	0.001^**^	

^#^International Restless Legs Scale, ^*^ANOVA, ^**^Repeated Measurement Test

In addition, a repeated measures test revealed that the mean score of sleep quality and the IRLS questionnaire differed significantly between the three assessments in all three groups: control, magnesium oxide intervention, and B6 intervention (*P* < 0.05). This data indicates that all three groups attained relative progress; however, the questionnaire scores of the two intervention groups were significantly higher than those of the control group. In contrast, the mean Pittsburgh quality of sleep and IRLS questionnaire scores in the magnesium oxide intervention group were considerably lower than in the B6 intervention group, and this difference was statistically significant (P 0.05). This indicates that magnesium oxide is more beneficial than B6 for treating RLS/WED (Table 2).

## Discussion

In this study, 75 RLS/WED patients with a mean age of 40.06 ± 8.6 years were randomly assigned to one of three groups: vitamin B6 intervention, magnesium oxide intervention, or a placebo group. 68% of this study’s participants were female. The majority of participants were married, college-educated homemakers. There were no differences between the three groups in terms of demographic factors. Also, the primary factors of the research (disease severity and sleep quality) did not change significantly across groups before the intervention.

Two months after receiving the intervention, the disease severity and the quality of sleep improved in all three groups. Nevertheless, both intervention groups outperformed the control group. This result is consistent with other research on this subject, such as the study conducted by Sinniah and Metta [10, 11].

Magnesium may have a role in the pathophysiology of RLS/WED since investigations have revealed that magnesium levels in the blood of RLS/WED patients are lower than those of controls. In this regard, the research of Yıldırım and Apaydın indicates that the zinc and magnesium levels of pregnant women with this illness are much lower than those of other women. In addition, they demonstrated a correlation between this and the intensity of the syndrome symptoms, such that the lower serum levels of magnesium and zinc in patients, the more severe the symptoms [8].

In contrast, the findings of the present investigation contradict the findings of the study of Taj. In this prospective case-control study including 600 pregnant women with RLS/WED, the researchers discovered that a drop in iron consumption and an increase in magnesium intake were related to an increase in syndrome symptoms [22]. The pathophysiology of the illness in pregnant women may differ from that of nonpregnant women, which is one of the causes for this divergence. However, Trenkwalder et al. proposed in a systematic review that there cannot be a solid conclusion about the effectiveness of magnesium in treating RLS/WED. They claim it is unclear if magnesium alleviates RLS/WED. Nor can it be determined which subset of patients may experience advantages [6]. In a comprehensive evaluation of pharmacological and non-pharmacological therapy for RLS/WED, Anguelova et al. do not believe magnesium to be a valuable medication for treating the symptoms of this condition [23].

However, other studies suggest that magnesium plays an important role in hundreds of metabolic reactions and muscle function [24, 25]. As magnesium deficiency leads to neuronal excitability and enhances neuromuscular transmission [26, 27]. Magnesium makes muscles relax more easily, which could be due to magnesium’s ability to block calcium, which helps regulate nerves and muscles, rather than allowing calcium to activate nerves [28]. If magnesium is low, calcium is not blocked and nerves become overactive, causing muscle contractions. Another study also showed that magnesium can improve insomnia caused by restless leg syndrome. Another pathway influenced by Mg is the serotoninergic system [29].

This study also showed that vitamin B6 (pyridoxine) helps alleviate the symptoms of this illness. Although the improvement in sleep complaints was not as great as in the magnesium group, it was significant when compared to the control group, which received just pramipexole. Vitamin B6 is equally helpful as propranolol in treating anxiety caused by antipsychotic medicines, according to certain studies. However, no research has been conducted on the efficacy of vitamin B6 in alleviating RLS/WED symptoms. Additionally,  some investigations have demonstrated that the B vitamin levels of these individuals are lower than those of the control group [30]. Pyridoxine is a covalently bound cofactor of glycogen phosphorylase. Phosphorylase is a major muscle protein and therefore represents a significant pool of pyridoxal phosphate. Pyridoxal phosphate is also important in carbohydrate metabolism as a cofactor of glycogen phosphorylase. A molecule of pyridoxal phosphate covalently bound to a lysine residue of each phosphorylase subunit is essential for the maintenance of enzyme activity and muscle function [31]. Vitamin B6 insufficiency is, therefore, one of the treatable etiologies of this disease. However, other researchers have also ruled out a connection between vitamin B6 levels and RLS/WED, concluding that the two are unrelated [32]. Therefore, further investigation is recommended.

## Conclusion

This study showed that magnesium and vitamin B6 administered for two months could lessen RLS/WED symptoms and enhance sleep quality in people with this condition. However, there is no clear cure for this disease, and it is not possible to expect a cure with these medications; however, magnesium and vitamin B6 supplements can be used in conjunction with other therapies to alleviate the symptoms of this disease. However, due to the discrepancy in the current information, further research is required in this field.

## Study limitations

This study was conducted on a few individuals who did not have a risk factor; therefore, the results should be extrapolated with caution to other patients. On the other hand, as this study was conducted blindly, the interpretation of the results may have been biased. Another limitation of this trial is that it was single-blind, so it may have influenced the interpretation of the findings.

## Data Availability

The datasets used and/or analysed during the current study are available from the corresponding author on reasonable request.
